# The Clinical Value of Long Noncoding RNA DDX11-AS1 as a Biomarker for the Diagnosis and Prognosis of Hepatocellular Carcinoma

**DOI:** 10.1155/2022/5735462

**Published:** 2022-08-29

**Authors:** Xiaojun Luo, Yang Wang, Xi Zhang, Wenbin Liu

**Affiliations:** Hepatic Biliary and Pancreatic Cancer Center, Chongqing University Cancer Hospital, Chongqing, China

## Abstract

Hepatocellular carcinoma (HCC) is a high-mortality malignant tumor with genetic and phenotypic heterogeneity, making predicting clinical outcomes challenging. The purpose of this investigation was to examine the potential usefulness of lncRNA DDX11 antisense RNA 1 (DDX11-AS1) as a biomarker for diagnosis and prognosis in hepatocellular carcinoma (HCC). The TCGA-LIHC datasets were searched for patients' clinical information and RNA-seq data, which were then collected. Relative expression levels of DDX11-AS1 in HCC tissues were determined by qRT-PCR. In order to test the sensitivity and specificity of the DDX11-AS1 receiver, receiver operating characteristic curves were utilized. The association of DDX11-AS1 expression with clinicopathological factors or prognosis was statistically analyzed. We found that the levels of DDX11-AS1 were higher in HCC specimens than in normal specimens. ROC analysis showed that DDX11-AS1 was a useful marker for discriminating HCC tissues from normal nontumor specimens. According to the results of clinical tests, a high level of DDX11-AS1 expressions was significantly related to the pathologic stage (*p*=0.015) and the histologic grade (*p* < 0.001). Survival studies indicated that patients with higher DDX11-AS1 expression had a significantly poorer overall survival (*p*=0.005) and progression-free interval (*p*=0.003) than those with lower DDX11-AS1 expression. Multivariate survival analysis verified that DDX11-AS1 expression level was an independent predictor for HCC patients. Overall, DDX11-AS1 may serve as a tumor promotor during HCC progression, and its high level may be a potential marker for HCC patients.

## 1. Introduction

Human hepatocellular carcinoma (HCC), which ranks one of the most common and aggressive hepatic illnesses, is the third most widespread cause of cancer-associated mortality around the world, in particular, in East Asia as well as sub-Saharan Africa [1]. The incidence rate has been increasing in China [2]. HCC makes up the larger part of the malignancy of the liver, which often results from the average clinical risk factors [3]. Surgical resection is regarded as the most effective therapy for HCC. However, about 80% of patients were diagnosed with locally advanced or metastasis tumor and were not suitable for hepatectomy [4]. Furthermore, it has been demonstrated that >45% of patients with HCC relapse in the follow-up time after resection [5]. The former research studies demonstrated a great number of HCC-related deregulated genes and signaling pathways; however, the highly complicated molecular mechanisms based on carcinogenesis and progressions are still less explicit [6, 7]. Thus, the identification of better underlying molecular markers for HCC is essential for more accurate early diagnosis and more effective therapeutic strategies.

As genome and transcriptome sequencing technologies are developing and genomics consortiums are being implemented, it has been demonstrated that a large proportion of the genome acts as an example for the transcribing of noncoding RNAs (ncRNAs) [8, 9]. lncRNAs are characterized as noncoding RNA molecules that are orientated with more than 200 nucleotides; they have the potential to play crucially essential roles in chromatin modification, regulation of transcription genes, and post-transcriptional management [10, 11]. More and more evidence has demonstrated that lncRNAs have been implicated in pathophysiological procedures like gene expression, cell multiplication, apoptosis, as well as tumor genesis [12, 13]. Recent years have witnessed that, as new technological methods are developing, great numbers of lncRNAs have been discovered to express a strong correlation with the dysregulation process of various cancers, which serve as oncogenes or tumor suppressors [14, 15]. Loss-of-function and gain-of-function experiments have led to the discovery of a number of lncRNAs with functional properties. Nonetheless, in spite of the discoveries, there are merely a small number of lncRNAs that have been characterized, and the functions featured by most lncRNAs have not been given characterization and are in need of in-depth investigation.

lncRNA DDX11 antisense RNA 1 (DDX11-AS1), located on 12p11.21, is a newly identified tumor-related lncRNA that has been reported to be dysregulated in several tumors, such as glioma and bladder cancer [16, 17]. In addition, the oncogenic roles of DDX11-AS1 have been demonstrated in gastric cancer and osteosarcoma [18, 19]. However, its expression and function in HCC remain unclear. In this study, we aimed to explore the potential of DDX11-AS1 used as a novel biomarker for HCC patients.

## 2. Patients and Methods

### 2.1. Data Sources

The RNA-seq data of 374 liver cancer samples used in the TCGA-LIHC research were obtained from the UCSC Xena website (https://xena.ucsc.edu/), which was also the source of the clinical information. The data were presented in FPKM format. The nontumor specimens in TCGA-LIHC (*n* = 50) and nontumor specimens in GTEx (*n* = 110) were both included in the RNA-seq data. The datasets for both of these types of samples were retrieved from the UCSC Xena data center.

#### 2.1.1. Differential Expression Analysis of DDX11-AS1 in HCC

Differential analysis of DDX11-AS1 was performed on HCC samples and normal samples obtained from TCGA and GTEx. In order to study the differences in the levels of DDX11-AS1 found in normal liver tissues and HCC tissues, Student's *t*-tests were carried out. By analyzing the receiver operating characteristic (ROC) curve and the area under the curve (AUC), researchers were able to determine whether or not DDX11-AS1 has the potential to identify HCC tissues from normal tissues.

### 2.2. Patients and Specimens

From July 2021 to January 2022, 12 HCC patients who underwent complete resection of the tumor in Chongqing University Cancer Hospital were subsequently enrolled in our study. No patient was given chemotherapy, radiotherapy, or immunotherapy prior to surgery. Two pathologists confirmed the HCC diagnosis. All tissue samples were frozen in liquid nitrogen following the surgical removal and then put into storage at −80°C until further use. Current research obtained approval from the Ethics Committee of Chongqing University Cancer Hospital; every patient had signed the written informed consent.

#### 2.2.1. Real-Time PCR

The extraction of total RNA was attained from tissue samples by applying TRIzol reagent (Invitrogen, China) according to the directions of the manufacturer. RNA was put under reverse transcription into cDNA in virtue of the Prime-Script™ one -step RT-PCR kit (TaKaRa, Kunming, Yunnan, China). The performance of QRT-PCR reactions was conducted in virtue of an ABI7600 System (Applied Biosystems, Pudong, Shanghai, China) and SYBR Green PCR Master Mix (Takara, Hangzhou, Zhejiang, China). The reaction program was 5 min at 95°C, followed by three-step reactions at 95°C/30 s, 60°C/30 s, and 72°C/10 s for 40 cycles. The normalization of transcription levels was made oriented with GAPDH expression. The relative amount of DDX11-AS1 was calculated using the equation 2^−ΔΔCt^. The experiments were implemented with three copies for each case. The PCR primers for DDX11-AS1 or GAPDH are expressed below: lncRNA DDX11-AS1 forward: 5′-CCTCTGCCTACAATACAAAAGTCA-3'; lncRNA DDX11-AS1 reverse: 5′- CAGGGTAAATGTACTTCAGCCAC-3'; GAPDH forward: 5′-CGGTCTCCTCTGACTTCAA-3'; GAPDH reverse: 5′-GGTGAGGGTCTCTCTCTTC-3'.

### 2.3. Statistical Analysis

The entire statistical analysis was completed via R program 4.0.2 (R Core Team, Massachusetts, USA) and SPSS13.0 for Windows (SPSS Inc., Chicago, IL, USA). Statistical analyses between the two groups were evaluated in virtue of two-tailed Student's *t*-test or chi-square test. For the purpose of distinguishing HCC specimens from normal nontumor tissues, ROC curves were developed. The probabilities of overall survival (OS) and progression-free interval (PFI) were calculated through the Kaplan–Meier methods, and the comparison was made applying the Log-rank Test. The Cox regression model was used for univariate and multivariate analyses. Two-tailed *p* values lower than 0.05 were regarded to be of statistical significance.

## 3. Results

### 3.1. Increased Expression of DDX11-AS1 in HCC Tissues

**Figure 1:**
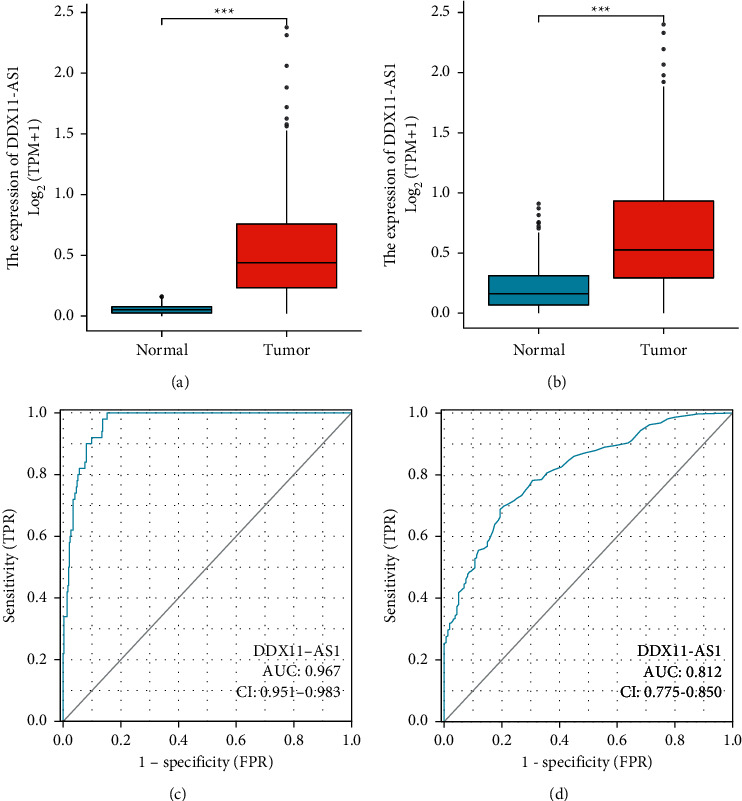
The expression of DDX11-AS1 and its diagnostic value in HCC patients. (a and b) DDX11-AS1 expression was distinctly increased in HCC specimens and nontumor specimens from TCGA datasets and/or GTEx data. (c and d) ROC assays for DDX11-AS1 as a diagnostic marker for HCC patients from TCGA datasets and/or GTEx data.

To investigate whether DDX11-AS1 was a functional lncRNA in HCC progression, our research explored the presentation of DDX11-AS1 in HCC and nontumor specimens from TCGA datasets. As presented in Figure 1(a), DDX11-AS1 expression was increased in HCC tissues in comparison to the nontumor tissues (*p* < 0.001). A similar result was also observed based on TCGA datasets and GTEx data (Figure 1(b)). After that, we investigated whether or not the levels of DDX11-AS1 had any diagnostic value. High DDX11-AS1 expressions yielded an AUC value of 0.967 (95 percent confidence interval: 0.951 to 0.983) for HCC in TCGA datasets, as indicated by the ROC assays (Figure 1(c)). In addition, high DDX11-AS1 expressions had an AUC value of 0.812 (95% CI: 0.775 to 0.850) for HCC in TCGA datasets and GTEx data (Figure 1(d)). According to the results of our research, DDX11-AS1 is a functional regulator in HCC.

### 3.2. DDX11-AS1 Associations with Clinical and Pathological Characteristics

To assess the clinical relevance harbored by DDX11-AS1 expressions in HCC, the median level of DDX11-AS1 performed the duty of a cutoff point to divide all 374 patients into two groups (high groups: *n* = 187 and low groups: *n* = 187). Subsequently, our group investigated the connection between the presentation of DDX11-AS1 and clinicopathological parameters. Based on Table 1, high expression of DDX11-AS1 was observed to be distinctly relevant to pathologic stage (*p*=0.015) and histologic grade (*p* < 0.001). Nevertheless, other characteristics did not show any significant difference.

### 3.3. Prognostic Significance of DDX11-AS1 Expression as a New Marker in HCC

**Figure 2:**
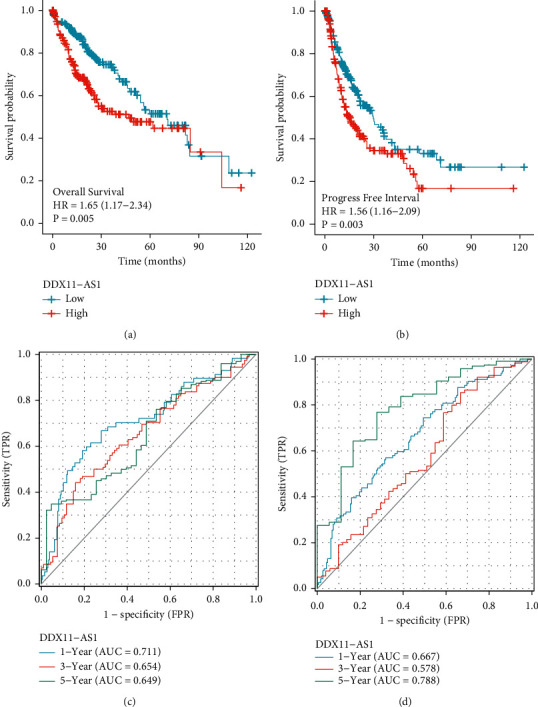
The significance of DDX11-AS1 expression as a predictive factor in patients with HCC. (a and b) Kaplan–Meier curves for OS and PFI and DDX11-AS1 expression in group of 374 HCC patients. (c and d) The AUC for 1-, 2-, and 3-year OS and PFI in TCGA datasets.

The Kaplan–Meier assays helped corroborate the link between the expression of DDX11-AS1 and the outcomes of 374 HCC patients. We observed that patients with high expression of DDX11-AS1 had poorer OS (*p*=0.005, Figure 2(a)) and PFI (*p*=0.002, Figure 2(b)) compared with those in the low DDX11-AS1 group. In addition, the areas under the time-dependent ROC for OS of the TCGA cohort are 0.711, 0.654, and 0.649 for 1-, 3-, and 5-year survival, respectively (Figure 2(c)). Moreover, the areas under the time-dependent ROC for PFI of the TCGA cohort are 0.667, 0.578, and 0.788 for 1-, 3-, and 5-year survival, respectively (Figure 2(d)). In addition, multivariate analyses were performed to establish whether or not DDX11-AS1 was a factor that was independent in the prognostic prediction of HCC patients. Importantly, the data revealed that high DDX11-AS1 expression can independently predict the clinical outcome of patients regarding OS (HR = 0.578, 95% CI: 0.398–0.838, *p*=0.004, Table 2) and PFI (HR = 0.615, 95% CI: 0.452–0.837, *p*=0.002, Table 3).

### 3.4. The Expression of DDX11-AS1 and Its Diagnostic Value in Our Cohort

**Figure 3:**
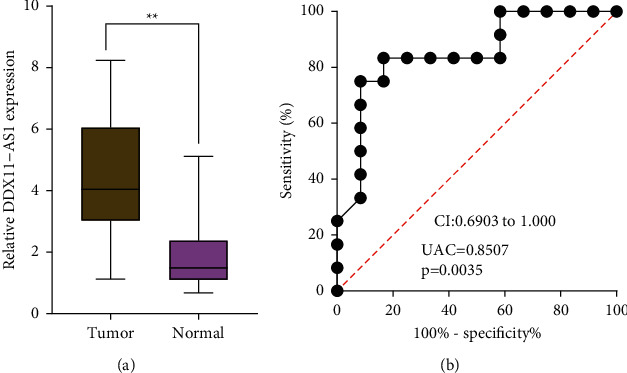
Identification of the expression of DDX11-AS1 in our cohort. (a) Relative expressions of DDX11-AS1 in HCC specimens and adjacent nontumor specimens determined using qRT-PCR. (b) ROC assays for DDX11-AS1 as a diagnostic marker for HCC patients.

To demonstrate the above results, we collected a total of 12 HCC samples and matched noncancerous tissue samples. The results of RT-PCR revealed that DDX11-AS1 expression was distinctly increased in HCC specimens compared with nontumor specimens (Figure 3(a)). After that, we investigated whether or not the levels of DDX11-AS1 had any diagnostic value. The ROC tests revealed that high DDX11-AS1 expression had an AUC value of 0.8507 (95 percent confidence interval: 0.6903 to 1.000) for HCC (Figure 3(b)). Our findings were consistent with the results from TCGA datasets.

## 4. Discussion

HCC features violence, invasion ability, particularly intrahepatically, and likely postresection recurrence [20]. Up to date, the clinical results featured by HCC patients have obtained no benefit from the latest progress of novel diagnostic and therapeutic approaches due to the supremely high recurrence rate and metastasis rate [21, 22]. Recent years have witnessed increasingly growing research studies which have unveiled the potential of lncRNAs used as novel diagnostic and prognostic markers for HCC patients due to their abnormal levels and the development of the wide detection of lncRNAs in the serum and tissues of tumor patients using high throughput sequencing [23, 24]. In addition, more than one lncRNA has been identified for positive relevance to the clinical outcome of HCC patients, such as lncRNA MCM3AP-AS1, lncRNA CASC9, and lncRNA-PDPK2P [25–27].

lncRNAs represent an emerging group, which may regulate HCC cell proliferation, migration, and apoptosis. For instance, lncRNA F11-AS1 was shown to be highly expressed in HCC and its overexpression suppressed the proliferation, migration and invasion of HCC cells, yet induced apoptosis via modulating NR1I3 through binding to miRNA-211-5p [28]. lncRNA HCG11 was found to be overexpressed in HCC and promote the proliferation and metastasis of HCC cells via the modulation of miRNA-26a-5p/ATG12 axis [29]. Those discoveries revealed that different lncRNAs might display a different part in HCC. Recently, Zheng et al. reported that the expressions of DDX11-AS1 were shown to be increased in glioma tissues and cells. There was a correlation between high levels of DDX11-AS1 expression and a poor prognostic value. DDX11-AS1 knockdown, from a functional standpoint, inhibited proliferation, migration, and invasion while simultaneously inducing apoptosis through the regulation of the miR-499b-5p/RWDD4 Axis [30]. Chen and his group showed that the expression of DDX11-AS1 was found at an astonishingly high level in bladder cancer and contributed to the aggressiveness of the disease. The proliferation was inhibited when DDX11-AS1 was knocked out through the mechanism of protecting LAMB3 from downregulation by sponging miRNA-2355-5p [16]. The above results revealed DDX11-AS1 as an oncogenic lncRNA in bladder cancer and glioma. However, the potential effects of DDX11-AS1 in HCC have not been explored.

This thesis first illustrated the distinct up-regulation of DDX11-AS1 expression in HCC specimens in comparison to nontumor specimens in TCGA datasets and our cohort, which was consistent with the expressing trend of DDX11-AS1 in bladder cancer and glioma. Further tests of the diagnostic usefulness of DDX11-AS1 confirmed that high DDX11-AS1 expression in the tumor specimens enabled the classification of HCC patients from normal specimens, suggesting that it is a viable diagnostic biomarker for HCC. Moreover, high DDX11-AS1 expression was observed to be of association with Pathologic stage and Histologic grade. A clinical study with a five-year following-up pointed out that patients who had high DDX11-AS1 expressions displayed a shorter OS and PFI, suggesting that overexpression of DDX11-AS1 may positively influence the clinical progression of HCC. In multivariate assays, increased DDX11-AS1 presentation was demonstrated to be an independent poor prognostic factor for both OS and PFI, indicating that DDX11-AS1 might be a promising biomarker for the diagnosis and prognosis of HCC patients. However, the small sample size of the present study was a limitation, which might likely lead to a not very convincing conclusion. In addition, whether the overexpression or knockdown of DDX11-AS1 may influence the proliferation and metastasis of HCC cells needed to be further studied in vitro and in vivo. In the future, we will collect more HCC specimens to further confirm our findings. Besides, the potential function of DDX11-AS1 will be further studied.

## 5. Conclusions

Our findings revealed that upregulation of DDX11-AS1 in HCC has an association with aggressive progression with poor prognosis and that DDX11-AS1 may function as a prognostic and diagnostic marker for HCC.

## Figures and Tables

**Table 1:** Association between DDX11-AS1 expression level and clinical characteristics.

Characteristic	Low expression of DDX11-AS1	High expression of DDX11-AS1	*p*
N	187	187	

Gender, *n* (%)			0.269
Female	55 (14.7%)	66 (17.6%)	
Male	132 (35.3%)	121 (32.4%)	

Age, *n* (%)			0.196
≤60	82 (22%)	95 (25.5%)	
>60	105 (28.2%)	91 (24.4%)	

Pathologic stage, *n* (%)			0.015
Stage I	97 (27.7%)	76 (21.7%)	
Stage II	35 (10%)	52 (14.9%)	
tage III	35 (10%)	50 (14.3%)	
Stage IV	4 (1.1%)	1 (0.3%)	

Histologic grade, *n* (%)			<0.001
G1	37 (10%)	18 (4.9%)	
G2	104 (28.2%)	74 (20.1%)	
G3	41 (11.1%)	83 (22.5%)	
G4	2 (0.5%)	10 (2.7%)	

Age, median (IQR)	62 (54, 70)	60 (51, 68)	0.056

**Table 2:** Univariate and Multivariate analyses of prognostic parameters for overall survival of patients with HCC.

Characteristics	Total (N)	Univariate analysis		Multivariate analysis	
		Hazard ratio (95% CI)	*p* Value	Hazard ratio (95% CI)	*p* Value
Gender	373				
Female	121	References			
Male	252	0.793 (0.557–1.130)	0.200		

Age	373				
≤60	177	References			
>60	196	1.205 (0.850–1.708)	0.295		

Histologic grade	368				
G1&G2	233	References			
G3&G4	135	1.091 (0.761–1.564)	0.636		

Pathologic stage	349				
Stage I&Stage II	259	References			
Stage III&Stage IV	90	2.504 (1.727–3.631)	<0.001	2.491 (1.717–3.615)	<0.001

DDX11-AS1	373				
High	186	References			
Low	187	0.604 (0.427–0.856)	0.005	0.578 (0.398–0.838)	0.004

**Table 3:** Univariate and Multivariate analyses of prognostic parameters for progression-free survival of patients with HCC.

Characteristics	Total (N)	Univariate analysis		Multivariate analysis	
		Hazard ratio (95% CI)	*p* Value	Hazard ratio (95% CI)	*p* Value
Gender	373				
Female	121	References			
Male	252	0.982 (0.721–1.338)	0.909		

Age	373				
≤60	177	References			
>60	196	0.960 (0.718–1.284)	0.783		

Histologic grade	368				
G1&G2	233	References			
G3&G4	135	1.152 (0.853–1.557)	0.355		

Pathologic stage	349				
Stage I&Stage II	259	References			
Stage III&Stage IV	90	2.201 (1.591–3.046)	<0.001	2.252 (1.625–3.122)	<0.001

DDX11-AS1	373				
High	186	References			
Low	187	0.642 (0.479–0.860)	0.003	0.615 (0.452–0.837)	0.002

## Data Availability

The data used in this research are available from the corresponding author upon reasonable request.
